# Modulation of natural killer cell functions by interactions between 2B4 and CD48 in *cis* and in *trans*

**DOI:** 10.1098/rsob.160010

**Published:** 2016-05-25

**Authors:** Maren Claus, Sabine Wingert, Carsten Watzl

**Affiliations:** Leibniz Research Centre for Working Environment and Human Factors at TU Dortmund(IfADo) Dortmund, Germany

**Keywords:** natural killer cells, 2B4, CD48, surface receptors, cytotoxicity

## Abstract

SLAM-related receptors (SRRs) are important modulators of immune cell function. While most SRRs are homophilic, 2B4 (CD244) interacts with CD48, a GPI-anchored protein expressed on many haematopoietic cells. Here we show that natural killer (NK) cell-expressed 2B4 not only binds in *trans* to CD48 on neighbouring cells but also interacts in *cis* with CD48 on the same cell. 2B4 uses the same binding site to interact with CD48 in *cis* and in *trans* and structural flexibility of 2B4 is necessary for the *cis* interaction. Furthermore, the *cis* interaction is sufficient to induce basal phosphorylation of 2B4. However, *cis* interaction reduces the ability of 2B4 to bind CD48 in *trans*. As a consequence, stimulation-dependent phosphorylation of 2B4 upon binding to CD48 positive target cells is reduced. Interfering with the *cis* interaction therefore enhanced the lysis of CD48-expressing tumour cells. These data show that the density of 2B4 and CD48 on both the NK cell and the potential target cell modulates NK cell activity.

## 1. Introduction

Natural killer (NK) cells are innate lymphoid cells and are important for effective early immune responses against viral infections and tumour formation. Through the engagement of activating receptors, NK cells are able to recognize and selectively kill transformed or virally infected cells [1]. Furthermore, they can secrete various cytokines and chemokines and are involved in modulating adaptive immune responses [2].

The family of SLAM-related receptors (SRRs) has important functions in modulating the reactivity of various immune cells [3]. Human NK cells express the SRRs NTB-A, CRACC and 2B4 [4]. While all other SRRs are homophilic, 2B4 recognizes the GPI-anchored Ig-like protein CD48 that is expressed on all haematopoietic cells including NK cells [5,6]. Binding of CD48 to 2B4 induces the phosphorylation of four immunoreceptor tyrosine-based switch motifs (ITSMs) in its cytoplasmic tail and recruitment of small adapter proteins SAP and EAT-2 [7]. This in turn activates signalling cascades resulting in NK cell cytotoxicity and production of cytokines such as IFNγ and TNF-α. While SAP can bind to all four phosphorylated ITSMs, the third ITSM can additionally recruit the phosphatases SHP-1, SHP-2, SHIP and the inhibitory kinase Csk [7,8].

In resting NK cells, 2B4 has co-stimulatory functions and can increase the response of other activating receptors in a synergistic manner [9]. In IL-2 primed NK cells, triggering 2B4 alone is sufficient to induce NK cell effector functions [2]. In addition, 2B4 seems to play an important role for integrin activation and the induction of a high-affinity state of LFA-1 [10]. Finally, triggering of 2B4 by CD48-expressing target cells or antibody cross-linking induces a strong downmodulation of 2B4 expression levels on NK cells, which might be another mechanism for regulation of NK cell function [11,12].

The extracellular part of 2B4 comprises an N-terminal V-type Ig-like domain, which contains the binding interface for CD48 [13,14], and a membrane-proximal C2-type Ig-like domain. The two Ig-like domains are connected by a six amino acid linker, and a short stalk couples the C2-domain to the transmembrane segment [13,15].

Several surface receptors have been shown to interact with their ligand on the surface of the same cell. Such *cis* interactions have been demonstrated for several inhibitory NK cell receptors with their MHC ligands [16]. Especially, the functional relevance of *cis* interaction between mouse Ly49A and its ligand H-2D^d^ for NK cell function was extensively studied. The authors demonstrated that *cis* interaction is masking the receptor for interaction with ligands in *trans*, thereby reducing recruitment of the receptor to the immunological synapse [17,18]. Further, sequestration of Ly49A through *cis* interaction was shown to be necessary for NK cell education by reducing the suppressive effect of unengaged Ly49 receptor during maturation [19,20]. Besides Ly49 receptors, also the Ig-like proteins of the LILRB family were found to interact with MHC in *cis*. For the mouse homologue PIRB it was shown that this *cis* interaction is involved in the regulation of mast cell activity. In contrast to Ly49A, the PIRB-MHC class I *cis* interaction is supposed to generate tonic inhibitory signals by counteracting the activating FcεRI [21,22].

Here we describe the interaction of the activating NK cell receptor 2B4 with its ligand CD48 in *cis* and the necessity of structural flexibility for this interaction. Furthermore, we find that this *cis* interaction modulates 2B4 cell surface expression and baseline phosphorylation. Finally, we show functional consequences for 2B4 phosphorylation after contact with susceptible target cells and subsequent cytotoxicity.

## 2. Results

**Figure 1.**
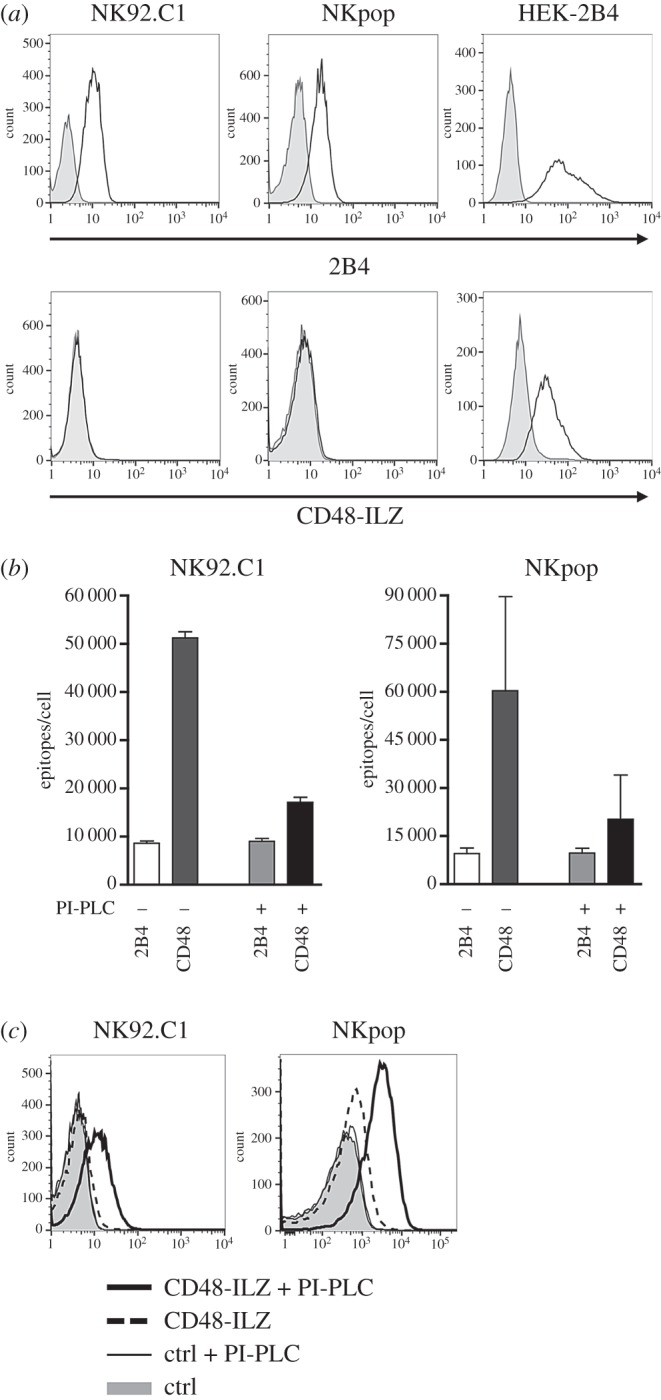
2B4 on NK cells is masked by *cis* interaction with CD48 on the same cell. (*a*) NK92.C1, primary NK cells and HEK-2B4 cells were stained for 2B4 using anti-2B4 mAb C1.7 (upper row) or soluble CD48-ILZ fusion proteins (lower row). Staining controls IgG1-PE or NKp30-ILZ (NK cells) and CS1-ILZ (HEK), respectively, are shown in grey. One representative experiment out of three is shown. (*b*) Absolute number of 2B4 and CD48 molecules on NK92.C1 and primary NK cells was determined before and after PI-PLC treatment using the QiFi kit. Data are shown as mean ± s.d. of three independent experiments. (*c*) NK92.C1 and primary NK cells were left untreated or treated with 1 U ml^−1^ PI-PLC for 1 h at 37°C. Cells were stained for 2B4 with soluble CD48-ILZ fusion proteins. NKp30-ILZ was used as negative control. One representative experiment out of at least three is shown.

Within the SRR family, 2B4 is the only heterophilic receptor and binds to the GPI-anchored protein CD48. To study the impact of this interaction on NK cell function, we investigated the binding of soluble CD48-ILZ fusion protein (sCD48) to 2B4 on primary NK cells and the NK cell line NK92.C1. While surface expression of 2B4 was clearly detectable by antibody staining (figure 1*a*, upper panel), we could not stain 2B4 on NK cells with sCD48 (figure 1*a*, lower panel). As a control, we achieved a clear staining of 2B4 on stably transfected HEK293T-2B4 cells using sCD48, demonstrating the functionality of this reagent. One difference between the HEK293T-2B4 and NK cells is that the latter also express CD48. Quantification of 2B4 and CD48 revealed that CD48 expression levels on NK cells exceed the number of 2B4 molecules by about fivefold (figure 1*b*). We assumed that a possible *cis* interaction between 2B4 and CD48 on the same NK cell might interfere with the binding of sCD48 in *trans*. To test this hypothesis, we treated NK92.C1 cells with phosphatidylinositol-specific phospholipase C (PI-PLC) to remove CD48 and all other GPI-anchored proteins from the cell surface. This resulted in a strong reduction of CD48 molecules per cell, while the number of 2B4 epitopes was not affected (figure 1*b*). Importantly, after PI-PLC treatment we now could detect the binding of sCD48 to NK cell 2B4 (figure 1*c*).

**Figure 2.**
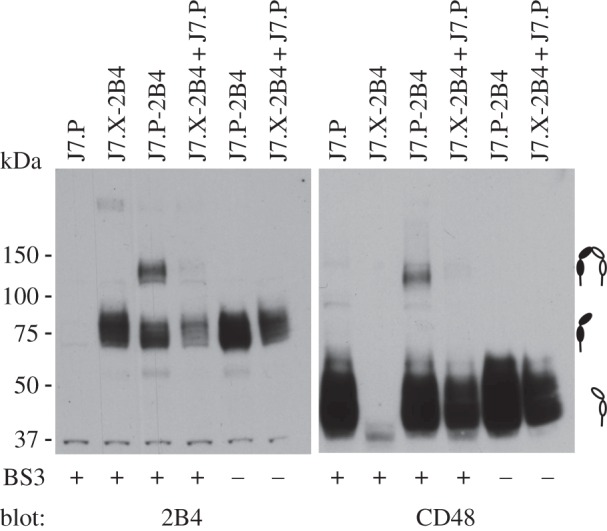
*Cis* interaction between 2B4 and CD48 on Jurkat cells. Jurkat J7.X and J7.P cells expressing CD48 and/or 2B4 were exposed to the chemical cross-linker BS^3^-D0. Cell lysates were analysed by reducing SDS-PAGE and western blotting. Membranes were probed with a biotinylated antibody against 2B4 (left panel) and reprobed to detect CD48 (right panel). 2B4 appears as prominent band at a size of 75 kDa (black symbol), CD48 is detected at 43 kDa (white symbol). After BS^3^ treatment an additional band of approx. 125 kDa appears only in the sample co-expressing 2B4 and CD48 on the same cell. One representative experiment out of four is shown.

To confirm the possible *cis* interaction between 2B4 and CD48, we took advantage of a Jurkat cell line defective in GPI-anchor synthesis. The J7.X cell line carries a mutation in the phosphatidylinositol glycan-A (*PIG-A*) gene and therefore does not express GPI-anchored proteins on the cell surface [23]. The J7.P cell line has been re-transfected with intact *PIG-A* cDNA and is therefore positive for GPI-anchored proteins. As all Jurkat cell lines are derived from CD4^+^ T cells, they do not express endogenous 2B4. With this cellular system, we were able to generate cell lines expressing either CD48 or 2B4 or both. To directly test for the interaction between 2B4 and CD48, we used the cell-impermeable chemical cross-linker bis(sulfosuccinimidyl)suberate (BS^3^-D0). Owing to a short spacer of 11.4 Å this cross-linker can only covalently link two proteins when they are in direct contact. We treated Jurkat cells expressing 2B4 (J7.X-2B4), CD48 (J7.P) or both (J7.P-2B4) either alone or in cell mixing experiments with the cross-linker and subsequently analysed the cell lysate by anti-2B4 and anti-CD48 western blotting (figure 2). In samples containing only 2B4-expressing cells, we detected a prominent band at 75 kDa, which corresponds to the expected size of fully glycosylated 2B4. This band was absent in lysates from untransfected Jurkat cells. Mature CD48 in J7.P cells was detected as a band of about 43 kDa. When 2B4 and CD48 were present in the same Jurkat cell (J7.P-2B4) an additional band of about 125 kDa was detected only when we treated the cells with the cross-linker. This band was detectable by anti-2B4 and anti-CD48 antibodies, suggesting that it represents a complex of 2B4 and CD48. This demonstrates that 2B4 and CD48 can interact not only in *trans* when present on different cells, but also in *cis* when both molecules are present on the same cell.

**Figure 3.**
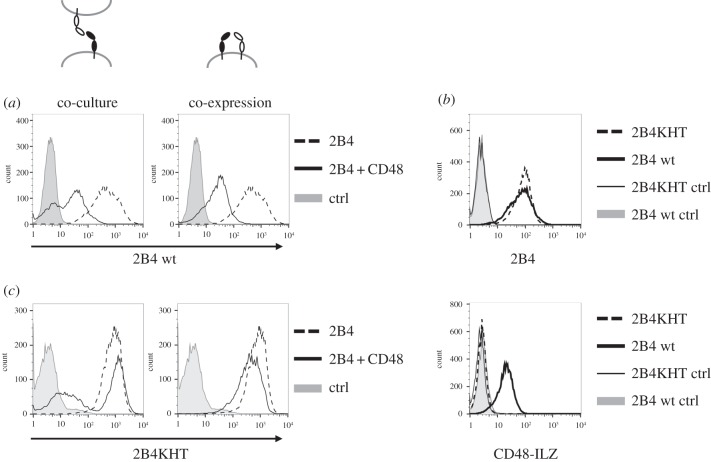
CD48 interaction in *cis* and *trans* modulates surface expression level of 2B4 in HEK cells via the same binding interface. (*a*) Left panel: HEK293T cells stably expressing 2B4 were mixed with equal amounts of HEK-CD48. 2B4 expression level was determined by flow cytometry after 24 h of co-culture using an anti-2B4 antibody. Right panel: HEK293T cells stably expressing 2B4 were transfected with pBABE-CD48 or the empty vector as a control; 24 h after transfection cells were detached and cultured for 24 h in a 75 cm^2^ flask without cell-to-cell contact. 2B4 expression levels were then determined by flow cytometry using an anti-2B4 antibody. Control staining (ctrl) with IgG1-PE is shown in grey. (*b*) HEK293T cells were stably transfected with pBABE-2B4wt or pBABE-2B4KHT. Cell surface expression of 2B4 was detected by anti-2B4 staining. Control staining (ctrl) with IgG1-PE is shown in grey (upper panel). Binding of CD48-ILZ fusion protein (lower panel) to the transfected cells was analysed by flow cytometry as described in figure 1. Control ILZ staining (ctrl) is shown in grey. (*c*) HEK293T cells stably expressing 2B4KHT were analysed for modulation through CD48 as described in (*a*). One representative out of at least three independent experiments is shown.

To investigate the structural requirements for this interaction in a more controllable system, we stably transfected HEK293T cells with vectors encoding 2B4 or CD48. We had previously shown that 2B4 is internalized when it is engaged by its ligand [11]. We could reproduce this finding when we engaged 2B4 in *trans* by mixing HEK cells expressing 2B4 with CD48-expressing cells, which resulted in a strong downmodulation of 2B4 surface expression (figure 3*a*, left). To investigate the co-expression of 2B4 and CD48 on the same cell, HEK-2B4 cells were transiently transfected with CD48 or empty vector. This also resulted in a decrease of 2B4 surface expression; however, under normal culture conditions we could not discriminate if this was due to the interaction between 2B4 and CD48 in *cis*, or due to the engagement of 2B4 by neighbouring CD48-expressing cells in *trans*. We therefore cultured the HEK-2B4 cells at a very low density to drastically limit cell contact and thereby prevent *trans* interactions. Also under these conditions we observed a downmodulation of 2B4 which was due to the presence of CD48 on the same cell (figure 3*a*, right). This indicates that the interaction between 2B4 and CD48 in *cis* also occurs in transfected HEK cells and that the *cis* interaction is sufficient for modulation of 2B4 surface expression.

Next, we wanted to investigate the structural basis for the 2B4/CD48 *cis* interaction. For this we wanted to use a 2B4 mutant that can no longer interact with CD48 in *trans*. In our hands, the previously published 2B4 mutant K68A/E70A [24] did not interfere with binding of sCD48 and showed no impairment in a functional assay (electronic supplementary material, figure S1). We therefore generated a new 2B4 binding defective mutant (2B4KHT) based on the published crystal structure of mouse 2B4 bound to its ligand [13]. The introduction of three alanine substitutions K54A, H65A and T110A within the putative ligand binding site completely disrupted binding of sCD48 to HEK cells stably expressing 2B4KHT (figure 3*b*). As a consequence, co-culture of HEK-2B4KHT with HEK-CD48 cells did not lead to downmodulation of 2B4 expression, confirming the elimination of the *trans* interaction (figure 3*c*). Interestingly, we also did not observe a downmodulation of 2B4KHT upon interaction with CD48 in *cis* when we co-expressed the mutant together with CD48 in the same cell and eliminated *trans* interaction by culturing the cells without cell-to-cell contact (figure 3*c*). From these findings, we conclude that *cis* and *trans* interaction with CD48 are mediated by the same binding epitope in 2B4.

The use of the same interface in 2B4 for both *cis* and *trans* binding implies either flexibility of the cell membrane, or intramolecular flexibility of 2B4 or CD48 in order to interact with neighbouring molecules. The intramolecular flexibility of 2B4 might be provided by the short linker between the two Ig-like domains or the stalk between the membrane-proximal Ig-like domain and the transmembrane segment. Therefore, we created 2B4 deletion mutants lacking the linker (Δ127–132) or the stalk motif (Δ211–223) and transiently transfected them into HEK293T cells. Unfortunately, both mutant 2B4 receptors were only poorly expressed at the cell surface (electronic supplementary material, figure S2) making functional analysis impossible. However, they were detectable in permeabilized cells, indicating that the stalk region and the linker domain are indispensable for correct folding or trafficking of 2B4 to the cell surface. In addition, we generated HEK293T cells stably expressing a deletion mutant lacking the entire membrane-proximal Ig-like domain of 2B4 (ΔIg, aa 141–210). Co-expression of CD48 in these cells led to a strong decrease in 2B4 surface expression comparable with that of 2B4 wt (electronic supplementary material, figure S3). Similar results were obtained when the membrane-proximal Ig-like domain of CD48 (ΔIg, aa 132–212) was also deleted, indicating that the membrane distal Ig-like domains are sufficient for *trans* and *cis* interactions between 2B4 and CD48.

**Figure 4.**
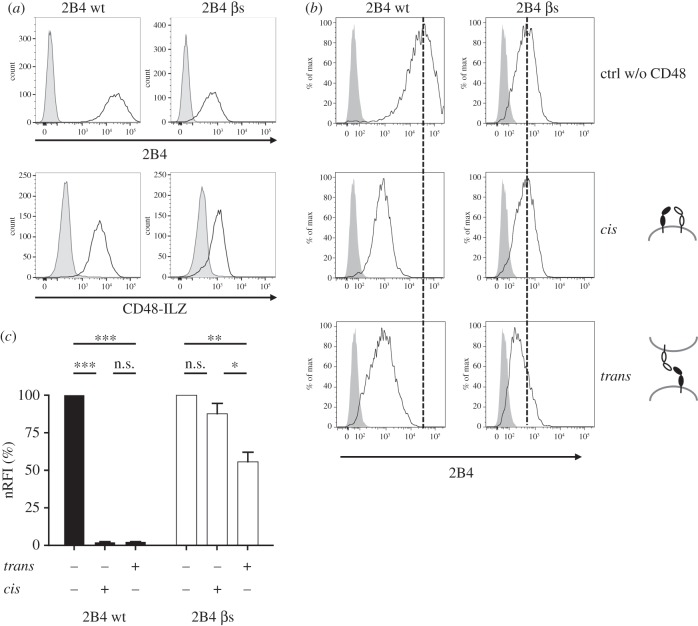
2B4 β-strand mutant does not interact with CD48 in *cis*. (*a*) HEK293T cells were stably transfected with pBABE-2B4wt or pBABE-2B4 βs. Cell surface expression (upper panel) and binding of CD48-ILZ fusion protein (lower panel) was analysed by flow cytometry. (*b*) HEK-2B4 wt and HEK-2B4 βs were analysed for modulation through CD48 as described in figure 3*a*. One representative experiment out of three is shown. (*c*) For statistical analysis the RFI was calculated from 2B4 MFI and normalized to 2B4 wt ctrl. Mean ± s.d. from data of three independent experiments are shown. ***p < 0.0001, **p = 0.0013, *p = 0.0306.

To explore the impact of the 2B4 linker on *cis* binding in more detail we exchanged linker aa residues 127–132 (DKVEKP) with the more rigid β-strand motif connecting the Ig-like domains D1 and D2 in human CD4 (114–127, QKEEVQLLVFGLTA) [25] to generate a more stiff 2B4 variant (2B4 βs). The 2B4 βs mutant was expressed on HEK293T cells, although to a lower extent than 2B4 wt. Furthermore, we could stain the 2B4 βs mutant with soluble CD48-ILZ, verifying the capability of 2B4 βs to interact with CD48 in *trans* (figure 4*a*). Additionally, co-culture of HEK cells stably expressing 2B4 βs with HEK-CD48 led to a considerable downmodulation of 2B4 βs surface expression similar to 2B4 wt (figure 4*b*,*c*). Importantly, co-expression of 2B4 βs and CD48 on the same cell did not affect the expression level of 2B4 βs (figure 4*b*,*c*), indicating that the stiffened linker between the Ig-like domains effectively interfered with the interaction of 2B4 and CD48 in *cis*. This demonstrates that the structural flexibility provided by the loop-like linker and the stalk motifs of 2B4 are sufficient to enable *cis* interaction between 2B4 and CD48.

**Figure 5.**
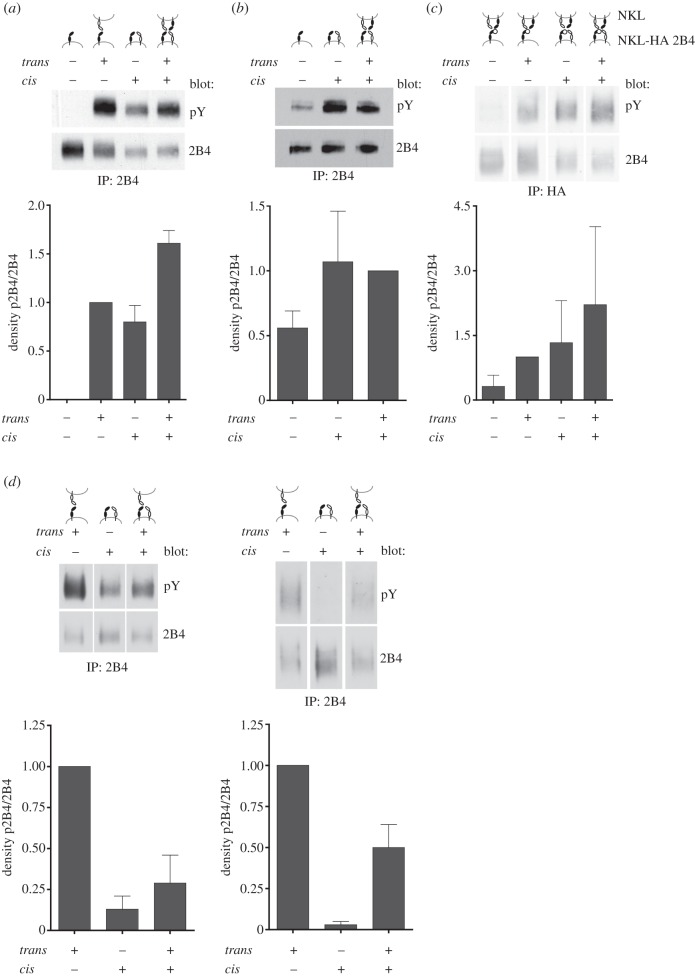
Regulation of 2B4 phosphorylation by *cis* and *trans* interaction with CD48. (*a*) Jurkat cells stably expressing 2B4 (black symbols) and/or CD48 (white symbols) were cultured for 2 h with or without cell-to-cell contact. 2B4 immunoprecipitates were analysed for tyrosine phosphorylation. Densitometric analysis was performed using ImageJ and p2B4 was calculated as pY density divided by total 2B4 density and normalized to *trans*. Data are presented as mean ± s.d. of two independent experiments. (*b*) NKL were treated with PI-PLC and cultured in the absence or presence of cell-to-cell contact. Then, cells were analysed for 2B4 phosphorylation as described in (*a*). 2B4 phosphorylation was calculated as pY density divided by total 2B4 density and normalized to ‘*trans* + *cis*’. Data are presented as mean ± s.d. of at least two independent experiments. (*c*) NKL HA-2B4 were treated with or without PI-PLC and cultured in the absence of cell-to-cell contact. Then, NKL HA-2B4 were mixed with normal NKL and stimulated either at 37°C or kept on ice. HA-2B4 immunoprecipitates were analysed for p2B4 as described in (*a*). 2B4 phosphorylation was calculated as pY density divided by total HA-2B4 density and normalized to *trans*. Data are presented as mean ± s.d. of at least two independent experiments. (*d*,*e*) NKL (*d*) or primary NK cells (*e*) were pre-treated with or without PI-PLC and cultured without cell-to-cell contact to establish *cis*-mediated baseline phosphorylation of 2B4. Cells were then mixed with Ba/F3-CD48 target cells to trigger 2B4 via CD48 in *trans*. 2B4 immunoprecipitates were analysed for tyrosine phosphorylation. Densitometric analysis of tyrosine phosphorylation relative to 2B4 was carried out as in (*a*). Data are shown as mean ± s.d. of three (NKL) or five (NKpop) independent experiments. Relative density of the *trans* sample was set to 1. For better comparability, blot lanes are displayed in the order of their appearance in the bar graphs. Original blots are shown in electronic supplementary material, figure S4.

Engagement of 2B4 results in rapid tyrosine phosphorylation of the ITSM motifs in its cytoplasmic part [26,27]. However, even in the absence of target cell contact we could detect some constitutive low level phosphorylation of 2B4 in NK cells [28]. We therefore speculated that this basal phosphorylation may be due to the *cis* interaction between 2B4 and CD48. To investigate this hypothesis we used the Jurkat cells expressing 2B4, CD48 or both molecules and analysed 2B4 phosphorylation. In cells expressing 2B4 and CD48, we readily detected a basal phosphorylation of 2B4, even when we only allowed *cis* interaction by culturing the cells without cell-to-cell contact (figure 5*a*). We detected a similar amount of 2B4 phosphorylation when we only allowed *trans* interaction between Jurkat-2B4 and Jurkat-CD48 cells. However, in the absence of CD48 we detected no 2B4 phosphorylation, demonstrating that the engagement of 2B4 by CD48 in *cis* or in *trans* between neighbouring cells is necessary for the basal phosphorylation of the receptor. To confirm this finding in NK cells we used the NK cell line NKL and reduced CD48 surface expression by PI-PLC treatment. Similar to our findings with Jurkat cells, *cis* binding of 2B4 to CD48 was sufficient to induce baseline 2B4 phosphorylation (figure 5*b*). As 2B4 is expressed by virtually all NK cells, it was difficult to assess the role of only the *trans* interaction between 2B4 and CD48. Therefore, we expressed HA-tagged 2B4 in NKL and manipulated CD48 expression by PI-PLC treatment. Similar to the Jurkat cells we could show in these NKL cells that *cis* interaction was sufficient to induce 2B4 phosphorylation to a similar extent as only the *trans* interaction, while we observed the strongest phosphorylation level when allowed for both interactions to occur (figure 5*c*). These data demonstrate that the *cis* interaction is sufficient to induce the basal phosphorylation of 2B4.

These results raised the question whether the degree of basal phosphorylation might affect the induced phosphorylation level that is caused by triggering of 2B4 in *trans* by CD48-expressing target cells. Therefore, NKL cells were pre-treated with PI-PLC and cultured with or without cell-to-cell contact to establish baseline phosphorylation levels. Subsequently, these cells were mixed with Ba/F3 cells expressing CD48 to trigger 2B4 in *trans*. As expected, cell mixing with susceptible target cells led to a further induction of 2B4 phosphorylation beyond baseline levels (figure 5*d*). Interestingly, the amount of induced 2B4 phosphorylation was lower in cells where 2B4 also interacted with CD48 in *cis*. Similar results were also obtained with freshly isolated primary human NK cells (figure 5*e*), suggesting that the *cis* interaction between 2B4 and CD48 can limit the *trans* interaction with CD48-expressing target cells and as a result reduce 2B4 phosphorylation induced upon contact with CD48-expressing target cells.

**Figure 6.**
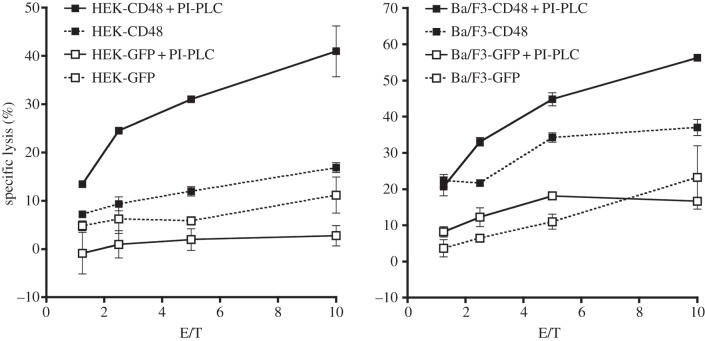
Modulation of NK cell cytotoxicity by 2B4–CD48 *cis* interaction. 2B4-mediated cytotoxicity of NK92.C1 was determined in a standard 4 h ^51^Cr release assay against CD48-expressing target cells. Data show one representative experiment out of at least three, each performed in triplicate.

We therefore addressed whether the *cis* interaction between 2B4 and CD48 has consequences for NK cell effector functions. Removal of CD48 by PI-PLC treatment increased the NK cell-mediated lysis of CD48-expressing HEK293T and Ba/F3 target cells (figure 6). However, PI-PLC treatment removes all GPI-anchored surface proteins, which could influence NK cell activity independently of 2B4. We therefore also tested for the lysis of CD48 negative, control transfected target cells. Importantly, this lysis was unaffected by the PI-PLC treatment, suggesting that the effect was specific for 2B4-mediated NK cell activation and was probably due to the removal of CD48 from the NK cells. This demonstrates that the *cis* interaction between 2B4 and CD48 on the surface of NK cells can limit the *trans* interaction of 2B4 and thereby modulate 2B4 engagement, phosphorylation and subsequently 2B4-mediated NK cell cytotoxicity.

## 3. Discussion

Our data show that 2B4 not only can bind to CD48 in *trans* but also interacts with CD48 in *cis* by using the same binding interface. As 2B4 is proposed to adopt a rod-like structure during interaction with CD48 in *trans* [13], the binding to its ligand on the same cell in *cis* requires large intramolecular rearrangements and implies great structural flexibility of the extracellular part of 2B4 and possibly also of CD48. Other receptors have been shown to interact with their ligands in *cis* and the flexible linker between Ig-like domains was proposed to be crucial for *cis* interaction of LILRs with MHC I [16]. Functional analyses and modelling of Ig-like domains of LILRB2 [29] and *Drosophila* Dscam [30] support our finding, that the short linker between the Ig-like domains of 2B4 is essential for providing structural flexibility to enable binding to CD48 in *cis* and in *trans*. In fact, our stiff 2B4 βs variant carrying the more rigid CD4 β-strand motif between the two Ig-like domains was defective in *cis* interaction, while the binding to soluble CD48 or CD48 on neighbouring cells remained intact. Deletion mutants lacking the entire membrane-proximal Ig-like domain of 2B4 or CD48 were still able to interact in *cis*. Therefore, the stalk regions of the surface molecules might provide additional flexibility to enable *cis* binding.

We demonstrated that constitutive phosphorylation of 2B4 ITSMs occurs only in the presence of CD48, and that *cis* binding is sufficient to induce substantial levels of baseline phosphorylation. We have previously shown that 2B4 is activation-dependently recruited to membrane microdomains and that this recruitment is essential for the phosphorylation of the receptor [28]. As the GPI-anchored CD48 is constitutively associated with membrane microdomains, its *cis* interaction with 2B4 might help to localize 2B4 into these specialized membrane domains and thereby induce receptor phosphorylation. Similar findings were described for a growing number of receptors. On B cells, *cis* binding of CD22 to sialic acids leads to phosphorylation of CD22 ITIMs and constitutive association with SHP1 [31,32]. This tonic inhibitory signalling is thought to increase the threshold for B-cell activation and to prevent unwanted/spontaneous B-cell activation [33,34]. Similarly, binding of mouse PIRB to MHC I on the same cell was shown to dampen accidental mast cell activation by constitutive association with SHP1, thereby increasing the threshold for activating signals [29]. These findings raise the question whether *cis* interaction of 2B4 can influence NK cell functions in a similar fashion. 2B4 can also have inhibitory functions in the absence of SAP [35] and we have previously shown that Csk, SHP1/2 and SHIP can bind to the third ITSM of 2B4 [8] to mediate these negative signals. However, so far, we were not able to show a correlation between *cis* interaction and association with inhibitory signalling molecules. Additionally, the interaction between 2B4 and CD48 may be important for some functions of CD48, as cross-linking of CD48 on mouse NK cells was shown to induce IL-13 production via co-clustering of 2B4 and subsequent signalling through 2B4 [36].

*Cis* binding of Ly49A to MHC I does not induce receptor phosphorylation [37], but instead reduces Ly49A accessibility for binding to MHC I ligand in *trans* by masking the receptor [18,37]. This sequestration of inhibitory Ly49A accessible in *trans* is thought to lower the threshold for activating signals. A growing number of receptors were shown to encounter their ligands in *cis*. Examples are the NK cell receptors NKp44 [38], Siglec7 [39] and herpesvirus entry mediator (HVEM) binding to BTLA (B and T lymphocyte attenuator) on T cells [40]. Similar to Ly49 receptors, *cis* binding does not induce receptor phosphorylation or downstream signalling, suggesting that modulation of activation threshold occurs by competitively inhibiting encounter of activating ligands in *trans*. Likewise, our data show that masking of 2B4 by NK cell CD48 abolishes binding of soluble CD48 and interferes with 2B4-mediated signalling during the encounter of CD48-expressing target cells. One might therefore consider a general role for *cis* interactions in the regulation of receptor function by modulating the threshold for receptor engagement in *trans*.

Along with its role in shaping NK cell activation threshold, Ly49 *cis* binding was also shown to impact the education of mouse NK cells [20]. Both *cis* and *trans* binding of Ly49A seem important for education, as Ly49 mutants only capable to interact in *trans* are not sufficient for education [41]. There is no evidence for *cis* binding of inhibitory NKG2/CD94 or KIR2DL1 [42], which are necessary for NK cell education in humans [43]. The signalling adapter SAP is absent in early NK cell development, resulting in inhibitory 2B4 functions [44,45]. It is, therefore, interesting to speculate that the 2B4 *cis* interaction may also play a role during NK cell development. PNH (paroxysmal haemoglobinuria) patients carry a somatic mutation in the *PIG-A* gene. Mosaicism of haematopoietic stem cells results in clonal expansion of blood cells lacking GPI-anchored proteins. A subset of NK cells from PNH donors lacks CD48. The resulting lack of 2B4 *cis* interaction may be one reason why these NK cells show a skewed KIR repertoire [46]. Additionally, reduced numbers of NK cells are found in the periphery due to defective chemokine function [47]. In functional assays, NK cells from PNH patients did not display defects in cytotoxicity [48]. However, these assays were performed with K562 target cells which lack CD48. Therefore, it is unknown how the absence of CD48 affects 2B4-mediated functions in these NK cells.

2B4 belongs to the family of SRRs. With the exception of 2B4 all of these receptors are homophilic [4]. As a consequence, any cell expressing a SRR also expresses the ligand for this receptor on its surface. It is, therefore, interesting to speculate that also these receptors engage in *cis* interactions. While it is difficult to experimentally distinguish between *trans* and *cis* interactions of homophilic receptors, it is likely that their function is also regulated by *cis* interactions on the surface of the same cell.

## 4. Material and methods

### 4.1. Reagents and cells

For flow cytometry the following antibodies (Abs) were used. PE-labelled anti-CD56 (MEM-188), FITC-conjugated anti-CD48 Ab (MEM-102), anti-2B4 (C1.7) labelled with FITC, APC or PE and PE-labelled donkey-anti-goat IgG were purchased from BioLegend. PE-labelled goat-anti-mouse IgG was purchased from Jackson ImmunoResearch. The anti-isoleucine zipper mAb (ILZ-11) [49,50] and rabbit-anti-2B4 [26] antibodies have been previously described. CD48-ILZ fusion proteins and respective negative controls NKp30-ILZ, NKp44-ILZ and CS1-ILZ were produced and purified as described previously [49,50].

The following antibodies were used for immunoprecipitation and western blotting. MOPC21 (Sigma), mouse-anti-2B4 (clone C1.7) and rabbit-anti-HA (clone C29F4, Cell Signaling Technology) were used for immunoprecipitation. Membranes were probed with biotinylated anti-phosphotyrosine (4G10, Upstate), polyclonal goat-anti-CD48 and biotinylated goat-anti-2B4 antibodies (both R&D Systems) and rabbit-anti-2B4 [26], and HRP-conjugated secondary antibodies goat-anti-rabbit, goat-anti-mouse, donkey-anti-goat or streptavidin (all Dianova/Jackson).

All media were purchased from Gibco, Life Technologies and were supplemented with 10% FCS and penicillin/streptomycin unless indicated otherwise. Polyclonal primary NK cells (NKpop) were purified from PBMC using the Untouched™ human NK cells kit (Invitrogen), according to the manufacturer's instructions. NK cells were between 90% and 99% NKp46^+^, CD3^−^ and CD56^+^, as confirmed by flow cytometry, and were cultured in IMDM, 10% human serum, 1% non-essential amino acids, 1% sodium pyruvate and 100 U ml^−1^ IL-2 (NIH cytokine repository), or IMDM, 10% FCS, penicillin/streptomycin and 100 U ml^−1^ IL-2.

Cell lines used were HEK293T cells, stably or transiently transfected with pBABEplus-CD48, pBABEplus-2B4 and empty pBABEplus vector, respectively, cultured in DMEM containing 0.5 µg ml^−1^ puromycin. For analysis of 2B4 surface expression, 2 × 10^5^ cells were cultured in 48-well plates (cell contact) or 75 cm^2^ flasks (no cell contact) for 8–24 h. The murine pre-B cell line Ba/F3 stably expressing CD48 was cultured in RPMI supplemented with 50 µM β-ME and 1 µg ml^−1^ puromycin. The GPI-deficient cell line Jurkat J7.X and the rescue cell line Jurkat J7.P [23] were a kind gift from Frank Momburg, DKFZ, Heidelberg, Germany. Cells were maintained in RPMI, J7.P were kept under selection with 750 µg ml^−1^ geneticin. Jurkat cells stably transfected with pMOW-2B4 were cultured with 0.5 µg ml^−1^ puromycin. The IL-2 independent NK cell line NK92.C1, stably expressing IL-2, was grown in alphaMEM containing 12.5% FCS, 12.5% horse serum, 1% penicillin/streptomycin and 50 µM β-ME.

### 4.2. Mutagenesis

Mutated variants of 2B4 and CD48 were generated by using standard PCR techniques as described elsewhere. The mutant 2B4 K68A E70A was generated using the primer published in Mathew *et al.* [24]. The following primers were used for mutagenesis.

**Table:** 

2B4 K54A for	5′ C AGC ATT GCA TGG GCG AAG TTG CTG 3′
2B4 K54A rev	5′ CAG CAA CTT CGC CCA TGC AAT GCT G 3′
2B4 H65A for	5′ GGA TTT CAT GCC ATA TTG AAG TGG G 3′
2B4 H65A rev	5′ C CCA CTT CAA TAT GGC ATG AAA TCC 3′
2B4 T110A for	5′ CTG GAG GTC GCC AGT ATA TCT GGA AAA G 3′
2B4 T110A rev	5′ C TTT TCC AGA TAT ACT GGC GAC CTC CAG 3′
2B4 + CD4β for	5′ CAG GTT TTT GTA TTT CAG AAG GAG GAG GTG 3′
2B4 + CD4β rev	5′ CAC CTC CTC CTT CTG AAA TAC AAA AAC CTG 3′
CD4β + 2B4 for	5′ GGA TTG ACT GCC CGC CTA CAG GGG 3′
CD4β + 2B4 rev	5′ CCC CTG TAG GCG GGC AGT CAA TCC 3′
2B4 del127–132 for	5′ GTT TTT GTA TTT CGC CTA CAG GGG CAG GGG 3′
2B4 del127–132 rev	5′ CCC CTG TAG GCG AAA TAC AAA AAC CTG GAA CG 3′
2B4 del Ig2 for	5′ CAG GGG AAG ATC CAG GAC TGT CAG AAT 3′
2B4 del Ig2 rev	5′ ATT CTG ACA GTC CTG GAT CTT CCC CTG 3′
CD48 del Ig for	5′ GTG CTT GAC CCT GTA CCA CCC TGT ACC CTG 3′
CD48 del Ig rev	5′ CAG GGT ACA GGG TGG TAC AGG GTC AAG CAC 3′
h2B4 del stalk long for	5′ CCT GAA TCT CAC TCC GTT TTT GGT GAT CAT CG 3′
h2B4 del stalk long rev	5′ G ATC ACC AAA AAC GGA GTG AGA TTC AGG GTG TG 3′

### 4.3. Flow cytometry

Flow cytometric analysis of intact and permeabilized cells using mAbs or ILZ fusion proteins was performed as described [49]. Quantitation of 2B4 and CD48 epitopes per cell was performed using the QiFi Kit (Dako) according to the manufacturer's instructions. All samples were measured on a FACSCalibur or LSR Fortessa and data were analysed using the FlowJo software (TreeStar, Inc.). The relative fluorescence index (RFI) for comparison of 2B4 expression levels was calculated by subtracting the mean fluorescence intensity (MFI) of staining with the control antibody from the MFI of the specific staining and dividing the result by the MFI of the control staining: RFI = (MFI specific − MFI control)/MFI control. Statistical significances were calculated with two-way ANOVA and Bonferroni's post test.

### 4.4. Chemical cross-linking

For chemical cross-linking, 2 × 10^6^ Jurkat cells per sample were washed twice with ice-cold PBS and were then resuspended in 250 µl PBS containing 0.7 mM bis(sulfosuccinimidyl)suberate (Pierce, Thermo Scientific). Samples were incubated for 30 min at 4°C. Reaction was quenched by addition of 20 mM (f.c.) Tris–HCl, pH 7.4. Cells were lysed in 50 µl lysis buffer (150 mM NaCl, 20 mM Tris–HCl, pH 7.4, 10% glycerol, 0.5% Triton X-100, 2 mM EDTA, 10 mM NaF) supplemented with 0.1% SDS, 0.5% Na-deoxycholate, 1 mM PMSF and 0.1 mg ml^−1^ DNase. Lysates were cleared by centrifugation and an equivalent of 0.5 × 10^6^ cells was analysed for 2B4 and CD48 by reducing SDS-PAGE and western blotting.

### 4.5. PI-PLC treatment

Cells were resuspended in medium at a concentration of 1 × 10^7^ cells ml^−1^ and incubated for 1 h at 37°C in the absence or presence of 1 U ml^−1^ PI-PLC (Sigma-Aldrich) and then washed with medium. Removal of CD48 was monitored by flow cytometry and cells were immediately used in functional assays.

### 4.6. Determination of 2B4 phosphorylation

For establishing 2B4 baseline phosphorylation, 1 × 10^7^ Jurkat cells per sample were incubated without cell-to-cell contact in a 175 cm^2^ flask or with cell-to-cell contact in a 9.6 cm^2^ well (6-well plate) for at least 2 h at 37°C. Then, cells were immediately put on ice and subjected to lysis and immunoprecipitation. Alternatively, PI-PLC-treated or untreated NKL cells (1 × 10^7^) were incubated without cell-to-cell contact in a 175 cm^2^ flask or with cell-to-cell contact in a 9.6 cm^2^ well (6-well plate) for 1 h at 37°C. Then, cells were chilled on ice, sedimented by centrifugation and lysed. 2B4 phosphorylation was analysed by immunoprecipitation and western blotting.

NKL HA-2B4 were treated with or without PI-PLC, washed and incubated for 30 min without cell-to-cell contact in a T175 flask to eliminate basal 2B4 phosphorylation, and then immediately put on ice. Pre-treated NKL HA-2B4 were mixed with an equal amount of normal NKL cells, centrifuged to establish cell-to-cell contact and incubated for 10 min either on ice or in a 37°C water bath. Incubation was stopped by addition of ice-cold PBS. Cells were lysed and HA-2B4 was immunoprecipitated.

To determine target cell-induced 2B4 phosphorylation, NKL or primary NK cells (1 × 10^7^) were treated with or without PI-PLC, washed and incubated for 30 min without cell-to-cell contact in a T175 flask to eliminate basal 2B4 phosphorylation, and then immediately put on ice. Pre-treated NK cells were mixed with (0.5 × 10^7^) Ba/F3-CD48, centrifuged to establish cell-to-cell contact and incubated for 10 min either on ice or in a 37°C water bath. Incubation was stopped by addition of ice-cold PBS. 2B4 phosphorylation was analysed by immunoprecipitation and western blotting.

### 4.7. Immunoprecipitation and western blotting

For immunoprecipitation, cells were lysed in lysis buffer (150 mM NaCl, 20 mM Tris–HCl, pH 7.4, 10% glycerol, 0.5% Triton X-100, 2 mM EDTA, 10 mM NaF) supplemented with 1 mM Na-orthovanadate, 1 mM PMSF. Pre-cleared lysates were first incubated for 1 h at 4°C with 0.5 µg of control IgG1 (MOPC21), followed by incubation with 0.5 µg of the indicated specific antibody, each coupled to 10 µl Protein G Dynabeads (Life Technologies). Beads were washed three times in cold lysis buffer and proteins were eluted in 2.5× reducing sample buffer (5% SDS, 25% glycerol, 12.5% 2-ME, 0.156 M Tris–Cl (pH 6.8), and 0.01% bromphenol blue). For western blotting, proteins were separated on 4–12% SDS NuPage gels (Life Technologies) and transferred to a PVDF membrane (Millipore). Membrane was blocked with 5% milk powder in PBS-T and incubated at 4°C overnight with the indicated primary antibodies. After washing, the membrane was incubated with the respective HRP-conjugated secondary antibody and developed using SuperSignal West Pico or Dura (Pierce).

### 4.8. ^51^Chromium release assay

Target cells were labelled in 100 µl assay medium (IMDM with 10% FCS and 1% penicillin/streptomycin) with 100 µCi ^51^Cr (Hartmann Analytik, Braunschweig, Germany) for 1 h at 37°C in a humidified 5% CO_2_ incubator. Cells were washed twice and resuspended at 5×10^4^ cells ml^−1^ in assay medium. Five thousand target cells/well were used in the assay. NK92.C1 were distributed on a U-bottom 96-well plate. Effectors were mixed with labelled target cells at different effector-to-target ratios. Maximum ^51^Cr release was determined by incubating target cells in 1% Triton X-100. For spontaneous release, targets were incubated without effectors in assay medium alone. Plates were incubated for 4 h at 37°C and supernatant was harvested. ^51^Cr release was measured in a gamma counter. Percentage specific release was calculated as [(experimental release − spontaneous release)/(maximum release − spontaneous release)] × 100. All samples were performed in triplicates.

## Supplementary Material

Supplementary Figures 1-4
