# Supplementary material for: Modulation of natural killer cell functions by interactions between 2B4 and CD48 in cis and in trans
Source: Open Biol. 2016 May 25;6(5):160010. doi: 10.1098/rsob.160010 (PMC4892432; doi:10.1098/rsob.160010)
Supplement: Supplementary Figures 1-4 [file rsob160010supp1.pdf]

Supplementary Information

Modulation of Natural Killer cell functions by interactions between 2B4 and CD48 in cis and in trans

Maren Claus, Sabine Wingert and Carsten Watzl

Leibniz Research Centre for Working Environment and Human Factors at TU Dortmund, IfADo, Dortmund, Germany, watzl@ifado.de

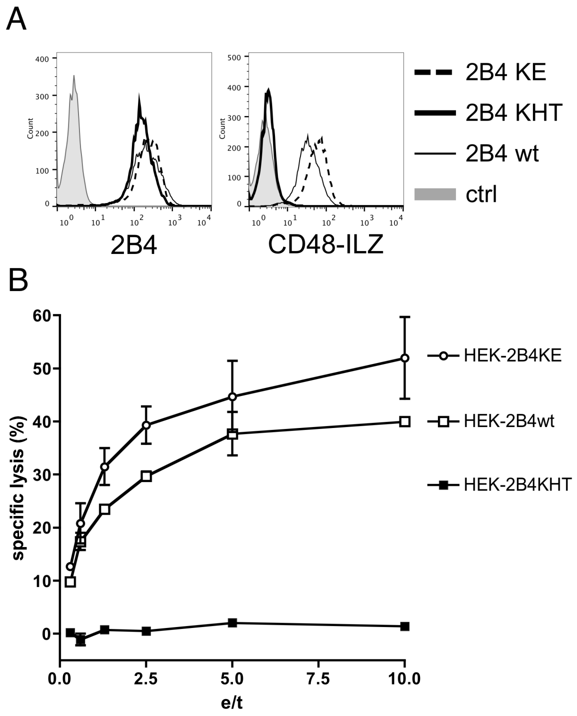

Figure S1: 2B4 KE mutant expressed on HEK cells does not affect ligand binding.

A. HEK293T cells were stably transfected with pBABE-2B4 wt, pBABE-2B4 KHT or pBABE-2B4 KE. 2B4 cell surface expression (left panel) and binding of CD48-ILZ fusion protein (right panel) was analysed by flow cytometry. CS1-ILZ was used as negative control. One representative of 2 independent experiments is shown.

B. NK92.C1 cells were used in a standard 4 h ^{51}Cr release assay against HEK293T cells stably expressing the indicated 2B4 molecules as described [1]. Data show one representative experiment performed in triplicate.

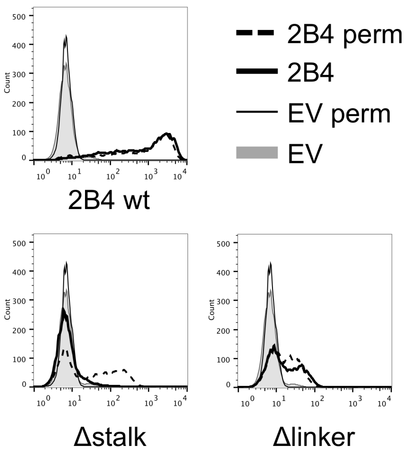

Figure S2: Linker and stalk domains are necessary for 2B4 surface expression.
HEK293T cells were transfected with pBABE-2B4wt, pBABE-2B4 Δstalk, pBABE-2B4 Δlinker or empty vector (EV). Cell surface expression was analysed by flow cytometry on intact and permeabilized cells. One representative of 3 independent experiments is shown.

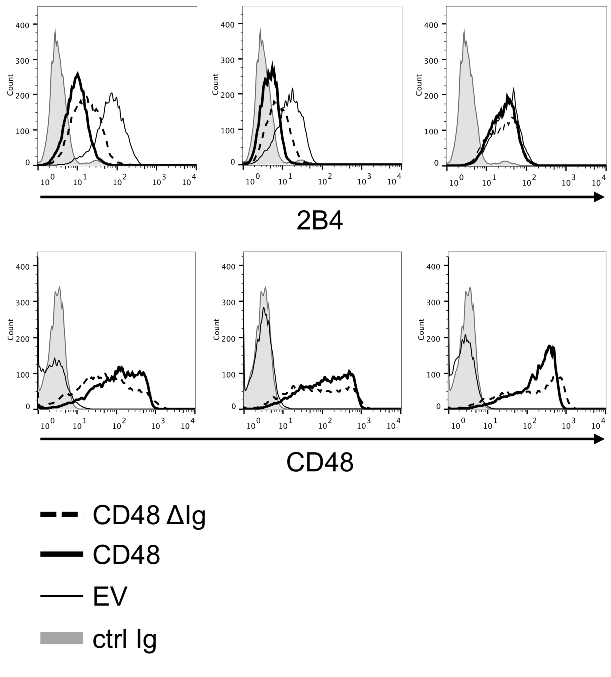

Figure S3: Membrane distal Ig-like domains of 2B4 and CD48 are sufficient for cis interaction.
HEK293T cells stably expressing 2B4 wt, 2B4 ΔIg or 2B4 KHT were transfected with pBABE-CD48, pBABE-CD48 ΔIg the empty vector (EV) as a control. 24 h after transfection cells were detached and cultured for 24 h in a 48 well plate to allow cell-to-cell contact. Expression of 2B4 and CD48 was analysed by flow cytometry. Control IgG staining is shown in grey. One representative of 3 independent experiments is shown.

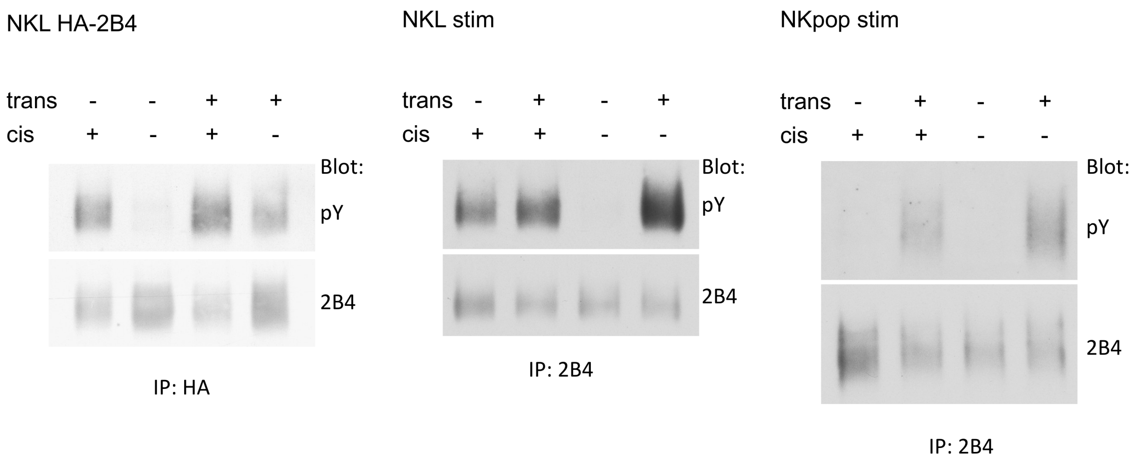

Figure S4: Original blots from figures 5C, D and E are shown.

References

1 Messmer, B., Eissmann, P., Stark, S., Watzl, C. 2006 CD48 stimulation by 2B4 (CD244)-expressing targets activates human NK cells. *Journal of immunology*. **176**, 4646-4650.
